# Comparison of the Expression of Acetylated Histones H3 and H4 and the Deacetylase Enzymes HDACs 1, 2, and 6 in Neoplastic and Nonneoplastic Canine Mammary Tissues

**DOI:** 10.1155/vmi/3876142

**Published:** 2025-06-23

**Authors:** Igor Luiz Salardani Senhorello, Oscar Rodrigo Sierra Matiz, Isabela Cristina Canavari, Giovanni Vargas-Hernandez, Letícia Abrahão Anai, Roberto Andrés Navarrete Ampuero, Josiane Moraes Pazzini, Cibele Maria Prado, Flávio Vieira Meirelles, Rosemeri de Oliveira Vasconcelos, Mirela Tinucci-Costa

**Affiliations:** ^1^Department of Veterinary Clinical Sciences and Surgery, School of Agricultural and Veterinary Sciences, São Paulo State University (UNESP), Jaboticabal, São Paulo, Brazil; ^2^Department of Clinical Sciences, Surgery and Animal Reproduction, School of Veterinary Medicine, São Paulo State University (UNESP), Araçatuba, São Paulo, Brazil; ^3^Department of Animal Health, National University of Colombia (UNAL), Bogotá, Colombia; ^4^Independent Veterinary Practitioner, Catanduva, São Paulo, Brazil; ^5^Independent Veterinary Practitioner, São José do Rio Preto, São Paulo, Brazil; ^6^Department of Veterinary Medicine, School of Animal Science and Food Engineering, University of São Paulo (USP), Pirassununga, São Paulo, Brazil

**Keywords:** canine, epigenetics, hypoacetylation, mammary neoplasia

## Abstract

Epigenetic alterations play a crucial role in the pathogenesis of cancer, as changes in the expression of DNA-associated proteins can affect gene expression. However, these changes may be reversible following treatment. This study aimed to evaluate the expression of acetylated histones H3 and H4 and the deacetylase enzymes HDACs 1, 2, and 6 in canine mammary tissues in order to identify potential alterations due to aberrant protein expression in neoplastic tissues. For this purpose, mammary tissue samples from 91 canine patients were divided into four groups: G1, control group composed of mammary tissues with no histopathological changes (*n* = 11); G2, simple mammary adenomas (*n* = 19); G3, simple mammary carcinomas without metastasis (*n* = 46); and G4, simple mammary carcinomas with lymph node metastasis (*n* = 15). The tissues were subjected to immunohistochemical analysis to assess protein expression. Antibody validation was performed by Western blot. The antibody expression results were evaluated semiquantitatively, considering the staining intensity and the percentage of marked cells. Univariate and multivariate analyses with a 5% significance level revealed differences in the expression of acetylated histones and deacetylase enzymes among the experimental groups (*p* < 0.05). Reduced acetylation of H3 (H3K9Ac) was observed in both nonmetastatic and metastatic simple mammary carcinomas compared to normal mammary tissue. Additionally, lower expression of HDAC1 and HDAC2 was found in neoplastic mammary tissues compared to normal tissue (*p* < 0.05). Conversely, HDAC6 exhibited higher expression in neoplastic mammary tissues (*p* < 0.05). There was no difference in the expression of acetylated H4 (H4K12Ac) among the groups (*p* > 0.05). Multivariate analysis showed a positive association between the expression of HDAC1 and HDAC2 and a negative association between H3K9Ac and HDAC6. These associations highlighted aberrant expression in mammary carcinomas compared to normal mammary tissues, indicating that epigenetic alterations exist in canine mammary neoplasms and that high HDAC6 expression may explain the observed hypoacetylation of H3 in neoplastic tissues. Collectively, these findings suggest that such alterations could potentially be therapeutic targets for the treatment of mammary cancer in dogs.

## 1. Introduction

Mammary tumors are among the most commonly diagnosed neoplasms in intact female dogs. They account for about 50% of all neoplasms in female dogs, with over 50% of these being malignant, highlighting their clinical significance in veterinary oncology [1–3]. These tumors exhibit striking biological and clinical similarities to human breast cancer, making them a valuable comparative model for studying tumor pathophysiology and potential therapeutic approaches [2]. Despite advancements in veterinary oncology, substantial variability in disease progression among patients with tumors of the same histological type, grade, and stage highlights the need for prognostic markers and more tailored treatment strategies [4, 5].

Surgical excision remains the primary treatment for canine mammary tumors; however, adjuvant therapies, such as chemotherapy, have produced inconsistent results regarding efficacy and long-term outcomes [6]. Prognostic assessment in veterinary oncology is typically grounded in clinical, pathological, and molecular parameters, but these factors alone do not always yield a comprehensive prediction of disease progression. The ongoing pursuit of novel biomarkers and prognostic tools is therefore essential for refining clinical decision-making and enhancing patient outcomes [1, 7].

Epigenetic modifications, which regulate gene expression without changing the DNA sequence, have emerged as critical players in tumor biology [8–10]. Among these modifications, DNA methylation, histone acetylation, and chromatin remodeling influence gene transcription and cellular phenotype. The dysregulation of these processes is increasingly recognized as a driver of oncogenesis, affecting tumor behavior and response to treatment [11, 12].

These histones and deacetylase enzymes were selected based on prior studies indicating their involvement in chromatin remodeling and gene silencing mechanisms in cancer. Specifically, acetylated histones H3 lysine 9 (H3K9Ac) and H4 lysine 12 (H4K12Ac) are common markers of transcriptionally active chromatin, and their hypoacetylation is frequently associated with gene repression and tumor progression [13, 14]. Histone deacetylase Classes 1 and II (HDAC1 and HDAC2) primarily localize to the nucleus, where they regulate gene expression through chromatin condensation and transcriptional repression [15–17]. In contrast, HDAC6 (Class IIb) is primarily cytoplasmic and is involved in the deacetylation of both histone and nonhistone proteins, which affects cell motility, protein stability, and cellular stress response [18, 19].

In human oncology, alterations in histone acetylation and HDAC activity have been extensively investigated, with studies showing that aberrant HDAC expression contributes to tumor progression by repressing tumor suppressor genes [20–23]. Consequently, HDAC inhibitors (HDACi) have gained attention as promising therapeutic agents that can modulate the tumor microenvironment and enhance clinical responses [9, 10, 24, 25].

Although epigenetic alterations have been well characterized in human breast cancer [12, 26, 27], studies in veterinary medicine remain limited [25, 28, 29]. However, emerging evidence suggests that histone acetylation patterns and HDAC expression may play a significant role in canine mammary tumors [30, 31]. Hypoacetylation of histones H3 and H4, along with aberrant expressions of HDACs 1, 2, and 6, appears to constitute a crucial aspect of human epigenetic dysregulation, and a similar trend seems to occur among animals [25, 31–33].

Given the challenges of predicting disease progression and identifying effective treatment strategies for canine mammary tumors, further investigation into epigenetic mechanisms is needed. This study aims to evaluate the acetylation patterns of histones H3 and H4, as well as the expression of HDACs 1, 2, and 6, in both neoplastic and nonneoplastic mammary tissues of female dogs. By clarifying potential epigenetic changes, this research seeks to aid in the identification of novel prognostic markers and therapeutic targets that may improve clinical management and outcomes for dogs with mammary tumors.

## 2. Materials and Methods

This study received approval from the Animal Experimentation Ethics Committee of the Faculty of Agricultural and Veterinary Sciences at UNESP-Jaboticabal (protocol no. 016384/17). All owners gave informed consent prior to their dogs' inclusion in the study.

### 2.1. Patient Selection and Sample Collection

The study included a total of 91 female dogs. Of these, 61 dogs were prospectively enrolled and diagnosed with simple mammary carcinomas (groups G3 and G4), regardless of breed and age. The remaining 30 samples (G1 and G2) were retrieved from the archival database of the Veterinary Pathology Department at the School of Agricultural and Veterinary Sciences at UNESP and consisted of 19 simple adenomas and 11 normal mammary tissues. All included dogs were entire females (not spayed).

These patients were examined at the Veterinary Obstetrics and Oncology Services of the Veterinary Hospital at the School of Agricultural and Veterinary Sciences, UNESP, Jaboticabal, Brazil. Clinical staging was conducted based on physical examinations, laboratory analyses, and imaging studies. The animals underwent complete clinical evaluation, including palpation of the mammary chain and regional lymph nodes, cardiopulmonary auscultation, abdominal palpation, and mucosal assessment. In addition, complementary imaging exams (three-view thoracic radiography and abdominal ultrasound) and laboratory analyses such as complete blood count (CBC), serum biochemistry, and urinalysis were performed.

Tumor samples were collected during unilateral total mastectomy, following the standard surgical protocol at the Veterinary Hospital. Regional lymph nodes (inguinal and axillary) were also removed for histopathological evaluation. Only one tumor per dog was analyzed, focusing on the most prognostically significant lesion when multiple nodules were present, following the criteria proposed by Cassali et al. [34] and Rasotto et al. [5]. Special subtype carcinomas, carcinomas in mixed tumors, and mammary sarcomas were excluded from the study [34].

Histopathological evaluation was employed to classify the samples into various experimental groups. Lymph node metastasis was ruled out based on histopathological examination, assessing for the presence of neoplastic epithelial cells [34].

All tissue samples were fixed in 10% buffered formalin for 24–48 h and then preserved in 70% ethanol until processing. Subsequently, the samples were paraffin-embedded and sectioned to a thickness of 3 μm for histopathological and immunohistochemical analyses. Hematoxylin and eosin (HE) staining was used for histopathological evaluation.

Dogs diagnosed with distant metastases at initial presentation were excluded, but those that developed metastases during follow-up remained in the study.

The samples were divided into four groups:

  Group 1 (G1): control group, consisting of 11 normal mammary gland samples.
  Group 2 (G2): 19 simple mammary adenoma samples.
  Group 3 (G3): 46 simple mammary carcinoma samples without lymph node metastasis at the time of diagnosis.
  Group 4 (G4): 15 simple mammary carcinoma samples with histologically confirmed regional lymph node metastasis.

### 2.2. Survival Analysis

Overall survival time (OST) was defined as the duration from diagnosis to death. For animals that remained alive at the end of the 3-year follow-up, survival data were censored. Updates on patient status were collected through telephone contact with owners and documented in medical files.

### 2.3. Validation of Antibodies Through Western Blot

The antibodies employed in this study were previously validated for canine mammary tissues using the Western blot technique, in accordance with the protocol established by Senhorello et al. [31].

### 2.4. Immunohistochemistry

Immunohistochemical analyses followed established protocols [31, 35] to evaluate the expression of H3K9Ac and H4K12Ac, along with the HDACs 1, 2, and 6.

Paraffin-embedded tissue sections (3-μm thick) were mounted on silanized slides, deparaffinized, and rehydrated using standard procedures [36]. Antigen retrieval was performed in a microwave oven for three cycles of 4 min each, followed by incubation with primary antibodies (detailed in Table 1). A commercial polymer-based detection system (Novolink DS, Leica Biosystems) was used, and reactions were counterstained with Harris hematoxylin and dehydrated in graded concentrations of alcohol and xylene before mounting.

Human mammary tissues, generously provided by the VETPAT laboratory (Campinas, São Paulo), served as reaction controls, with adjacent healthy mammary tissue used as positive controls for the neoplastic samples. Negative controls were processed by replacing primary antibodies with antibody diluents to verify specificity.

Immunohistochemistry was chosen for its ability to spatially localize protein expression in tissue architecture, which is essential for distinguishing nuclear from cytoplasmic patterns, particularly for HDAC isoforms. Additionally, it allows for semiquantitative analysis while preserving morphological context [37].

### 2.5. Evaluation of Immunohistochemical Staining

Nuclear staining of H3K9Ac, H4K12Ac, HDAC1, and HDAC2 was verified by the presence of diffuse brown staining within the nuclei of cells from various experimental groups, consistent with patterns observed in positive controls. Immunohistochemical assessment was conducted using a staining score protocol. Five random fields per sample were photographed at 400x magnification with a Novel microscope (BM2100) using a Bioptika camera (CMOS-HD). Manual counting of 100 cells per field was performed with ImageJ software (v. 1.44p) and the “Cell Counter” tool, followed by the calculation of arithmetic means in accordance with the methodology applied in previous studies in canine tissues [29, 31, 35]. Fields were categorized based on the percentage of positive cells (0 = negative; 1 = 1–25%; 2 = 26–50%; 3 = 51–75%; 4 = 76–100%) and staining intensity (0 = negative; 1 = weak; 2 = moderate; 3 = strong), independently assessed by two blinded observers. The final staining score was obtained by multiplying these values, and the results were utilized for statistical analyses [31, 35, 38].

For HDAC6, which shows cytoplasmic expression, a different quantification method was utilized. Positive staining was indicated by brown cytoplasmic coloring, with the total percentage of the positive area being calculated. Five random fields (400x) were analyzed, as previously described [31, 35, 39]. The total photographed area was measured manually, excluding noncellular regions. The positively stained area was then selected, thresholded, and quantified. The percentage of the positive area was calculated for each image and averaged across the five images from each sample.

### 2.6. Statistical Analysis

Comparisons of histone (H4K12Ac, H3K9Ac) and HDAC (HDAC1, HDAC2) expression among experimental groups (G1, G2, G3, and G4) were carried out using the Kruskal–Wallis test, followed by Dunn's post hoc test. For HDAC6, intergroup comparisons were conducted only, as it was measured as a percentage of marked cells instead of analyzed with histones within groups.

Multivariate statistical methods were applied to explore associations between histones and HDACs within and between groups. Hierarchical cluster analysis was used to identify subgroups within each experimental group, with identified subgroups randomly assigned as A or B. Principal component analysis (PCA) was conducted to determine interactions between subgroups and latent variables (PC1, PC2). Exploratory factor analysis was then used to explain data variance, with factors (F1, F2) identified based on eigenvalues and Varimax rotation. Variables with absolute loadings > 0.5 were considered relevant. Differences in factors between Subgroups A and B were evaluated using the Wilcoxon–Mann–Whitney test.

To differentiate the groups, H3K9Ac, HDAC1, HDAC2, and HDAC6 were chosen based on the highest factor loadings. ROC curves were generated using F1 and F2 values to evaluate specificity, sensitivity, and cutoff points for distinguishing between groups (G2, G3, and G4) and G1. Survival analysis was performed using Kaplan–Meier estimates, with group comparisons conducted using the log-rank test.

Statistical analyses were carried out using R and Statistica software, with a significance threshold set at 0.05.

## 3. Results

### 3.1. Immunoexpression of Acetylated Histones and Deacetylase Isoenzymes: Univariate Analysis

**Figure 1:**
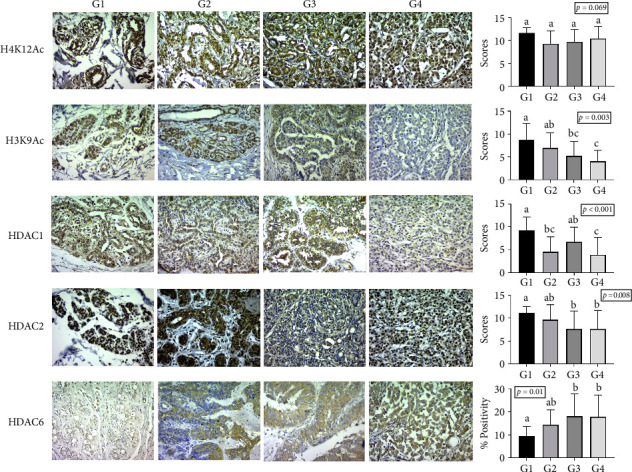
Photomicrographs depict the staining patterns for acetylated histones and HDAC isoenzymes. Graphs on the right summarize the mean staining scores (or positive area percentages for HDAC6) for each group. Different lowercase letters above the bars indicate statistically significant differences (*p* < 0.05) within the same marker. Statistical analysis was performed using the Kruskal–Wallis test followed by Dunn's post hoc test. Immunohistochemical staining was examined at X400 magnification.

Antibody staining was observed in all analyzed groups. When comparing the staining patterns with the positive control, similarities were noted as H3K9Ac, H4K12Ac, HDAC1, and HDAC2 exhibited predominantly nuclear staining, while HDAC6 showed predominantly cytoplasmic staining (Figure 1).

The Kruskal–Wallis test revealed significant differences (*p* < 0.05) in antibody scores for HDAC1, HDAC2, and H3K9Ac among the groups, as well as in the percentage of HDAC6 isoenzyme staining. However, no significant difference was noted for H4K12Ac antibody scores between the groups.

Statistical differences in H3K9Ac expression were specifically observed between groups G1 and G3 (*p*=0.022), G1 and G4 (*p*=0.002), and G2 and G4 (*p*=0.028), showing lower expression in malignant neoplasms. In terms of HDAC1 expression, differences were noted among groups G1 and G2 (*p*=0.002), G1 and G4 (*p* < 0.001), and G3 and G4 (*p*=0.010), with higher expression in normal mammary tissue compared to simple adenomas and metastatic carcinomas. Moreover, significant differences in semiquantitative HDAC2 expression were identified between groups G1 and G3 (*p*=0.007) and G1 and G4 (*p*=0.035), indicating lower HDAC2 expression in mammary carcinomas relative to normal tissue.

Lastly, HDAC6 immunostaining showed significant differences, with variations in the percentage of the positive area between groups G1 and G3 (*p*=0.005) and G1 and G4 (*p*=0.045). This isoenzyme demonstrated higher expression in mammary carcinomas compared to normal mammary tissues. The antibody staining results for groups G1, G2, G3, and G4 are summarized in Figure 1.

**Figure 2:**
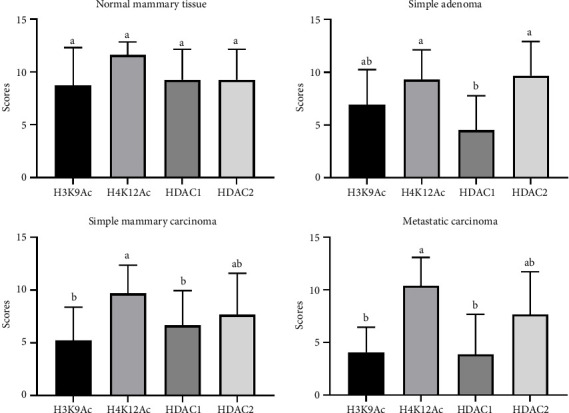
Graphical representation of staining scores for H4K12Ac, H3K9Ac, HDAC1, and HDAC2 in the groups: normal mammary tissue (G1), simple adenoma (G2), simple carcinoma (G3), and metastatic carcinoma (G4) in female dogs. Different letters indicate statistically significant differences (*p* < 0.05).

Univariate analysis within the experimental groups showed no significant differences in antibody expression in G1. However, in G2, HDAC2 expression was significantly higher than that of HDAC1 (*p* < 0.001), and H4K12Ac was expressed at higher levels than HDAC1 (*p* < 0.001). In G3, significant differences were noted between H4K12Ac and H3K9Ac (*p* < 0.001) as well as between H4K12Ac and HDAC1 (*p* < 0.001), while no significant differences were observed among the other antibodies. Likewise, in G4, H4K12Ac expression was significantly greater than that of H3K9Ac (*p* < 0.001) and HDAC1 (*p* < 0.001). Overall, in groups G3 and G4, H4K12Ac demonstrated higher expression levels compared to H3K9Ac and HDAC1 (Figure 2).

### 3.2. Immunoexpression of Acetylated Histones and Deacetylase Enzymes: Multivariate Analysis

**Figure 3:**
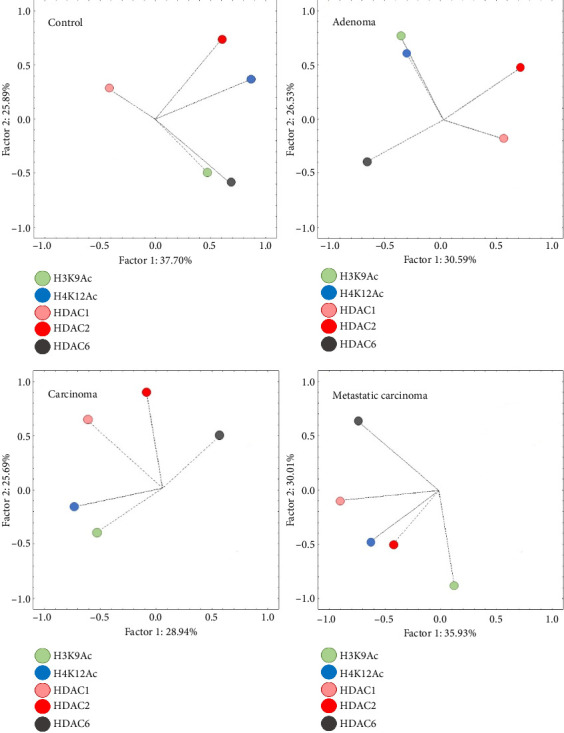
Multivariate principal component analysis for each experimental group based on the association between the expression of acetylated histones (H3K9 and H4K12) and histone deacetylase enzymes (HDAC1, HDAC2, and HDAC6). Colors represent different antibodies and are identified in the legend.

Exploratory factor analysis identified a positive association in Group G1 for the expression of H3K9Ac and HDAC6, while this association was negative in Groups G3 and G4. A similar association was noted between Groups G2 and G3 for H4K12Ac and H3K9Ac, as well as between Groups G3 and G4 for HDAC1 and HDAC2, both exhibiting a positive correlation. No consistent pattern of antibody expression association was observed between G1 and the other groups (Figure 3 and Table 2).

When comparing Subgroups A and B within each experimental group, Group G1 exhibited a significant difference between Subgroups A and B regarding the positively associated expression of H3K9Ac and HDAC6 (*p* < 0.001). In Group G2, a significant difference was noted between the subgroups for the negative association between HDAC1 and HDAC6 (*p*=0.005). In the analysis of the groups with mammary carcinomas, both groups G3 and G4 showed a significant difference between subgroups concerning the positive association of HDAC1 and HDAC2 (*p* < 0.05). These results indicate that within the malignant neoplasia groups, there are differing expression patterns, allowing for the dichotomization of Subgroups A and B (Table 2).

**Figure 4:**
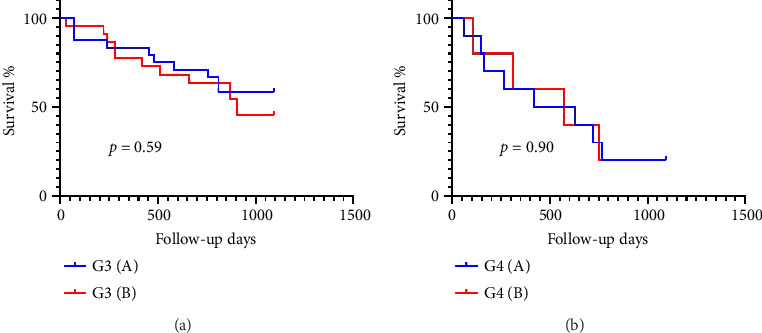
Kaplan–Meier survival curve for Subgroups A and B of Groups G3 (a) and G4 (b) based on the association between immunostaining identified through exploratory multivariate factor analysis.

After confirming the separation of samples into G3 and G4 based on the associated expression of histones, we evaluated whether this subdivision influenced prognosis. However, no significant difference in OST was observed between animals in Subgroups A and B of G3 or between Subgroups A and B of G4 (Figure 4).

Further multivariate analyses across the experimental groups identified two principal factors (Factor 1 and Factor 2), which accounted for 61.89% of the variance between groups. Factor 1 showed a positive association between HDAC1 and HDAC2 expression, while Factor 2 demonstrated a negative association between H3K9Ac and HDAC6 expression (Table 3).

ANOVA comparisons of these factors revealed significant differences among groups for Factor 1 (*p*=0.002) and Factor 2 (*p* < 0.001). Specifically, Factor 1 showed a significant difference between G1 and all other groups, while no significant differences were noted among the neoplastic groups. For Factor 2, significant differences were identified between G1 and G3, as well as between G1 and G4, but no differences were found among the neoplastic groups. These findings indicate a distinct expression pattern for the positive association between HDAC1 and HDAC2 in normal mammary tissue compared to neoplastic tissues, while the negative association between H3K9Ac and HDAC6 separated normal from malignant mammary tissues.

**Figure 5:**
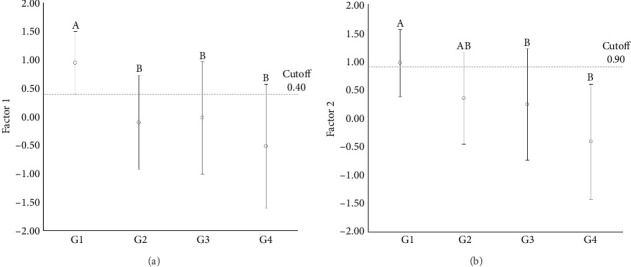
Graphical representation of cutoff points established by the ROC curve for Factor 1 (a) and Factor 2 (b) based on the exploratory factor analysis. Different letters indicate statistical differences (*p* < 0.05).

To further explore these differences, receiver operating characteristic (ROC) curve analyses were conducted to evaluate the sensitivity and specificity of these factors in distinguishing normal from neoplastic tissues. Among the key findings, a high sensitivity was noted for differentiating normal mammary tissue from simple adenomas (100%) and simple carcinomas (90.9%), as well as a high specificity for distinguishing normal mammary tissue from metastatic carcinomas (90.9%) for Factor 1. For Factor 2, a high specificity was observed in distinguishing normal mammary tissue from simple carcinomas (91.3%) and a high sensitivity was noted for differentiating normal mammary tissue from metastatic carcinomas (93.3%) (Table 3 and Figure 5).

## 4. Discussion

This is the first study to compare the expression of acetylated histones H3 and H4, as well as deacetylase enzymes HDAC1, HDAC2, and HDAC6, in normal, benign, and malignant mammary tissues in dogs. The results revealed distinct expression profiles among the groups, particularly highlighting a reduction in H3K9 acetylation and an increase in HDAC6 expression in malignant neoplasms. These findings support the notion that epigenetic alterations are involved in the pathogenesis of canine mammary tumors.

The staining patterns observed in canine mammary tissues were consistent with those described in previous studies on human mammary tissues, as well as in the control group tissues in the present study. Specifically, we noted predominantly nuclear staining for H4K12Ac, H3K9Ac, HDAC1, and HDAC2, along with cytoplasmic staining for HDAC6 [13, 40, 40].

Histone H3 hypoacetylation was notably observed in both nonmetastatic and metastatic simple carcinomas compared to normal and adenomatous tissues. This aligns with studies in human breast cancer, where hypoacetylation of H3K9 has been associated with tumor progression, probably by silencing tumor suppressor genes, resulting in poor prognosis [12, 13, 42]; Kouzarides, 2007. Similar patterns have also been reported in canine urothelial carcinoma [25], reinforcing the relevance of this marker in veterinary oncology.

Although the study did not consider prognostic factors, Senhorello et al. [31] found no effect of hypoacetylation on overall survival in female dogs, without assessing specific survival and disease-free time. As hypoacetylation is found in malignant mammary neoplasms, further research is warranted. Additionally, this modification may be a relevant focus for future therapeutic approaches.

In contrast, H4K12 acetylation did not differ significantly among groups. Although this mark remains conserved across neoplastic and nonneoplastic tissues in our study, other histone modifications such as K4K5, H4K16, and H4K8 may also play roles in gene regulation and deserve further investigation [14, 43, 44]. Furthermore, research indicates that hypoacetylation tends to begin with H4K16, progressing to H4K12, and subsequently to other lysine residues as tumor progression advances [14].

The absence of global alterations in H4K12Ac creates opportunities for studies evaluating other acetylation marks in canine mammary tumors. In humans, H4K12 acetylation alterations are primarily linked to tumor grade, a variable not analyzed in our study. More significant changes were observed in H4K16 acetylation, which is hypoacetylated and correlated with several unfavorable prognostic factors [13].

Among histone deacetylases, HDAC1 and HDAC2 (Class I) showed reduced expression in carcinomas compared to normal tissues. These enzymes have been extensively studied in human cancers, often with conflicting results. While some studies associate their overexpression with poor prognosis, others report decreased expression in aggressive tumors [16, 17, 24, 27, 45]. Our findings support the latter, suggesting that loss of HDAC1 and HDAC2 may be associated with tumor progression in dogs, although no association with survival time was observed in this cohort.

The observation of altered HDAC1 expression, particularly its reduced levels in aggressive tumors, may indicate that low HDAC1 expression serves as a negative prognostic factor. This hypothesis requires further studies for confirmation and a better understanding of this alteration. However, in a previous study, we found an association between lower HDAC1 expression and the presence of lymph node metastases in dogs with mammary tumors [31, 35].

Conflicting results for HDAC2 expression in canine mammary tumors suggest that the expression may vary between dogs and humans [16, 27, 38]. Furthermore, many studies failed to include comparisons between normal and neoplastic tissues, complicating the ability to draw definitive conclusions regarding these differences.

Conversely, HDAC6 (Class IIb) demonstrated increased expression in carcinomas relative to normal tissue. Notably, multivariate analysis revealed a negative association between HDAC6 and H3K9Ac in neoplastic tissues, suggesting that HDAC6 may play a direct role in H3 deacetylation in canine mammary carcinomas. These findings are consistent with previous research, indicating that HDAC6 overexpression contributes to histone hypoacetylation and tumorigenesis [18, 46].

The combined expression patterns of HDAC1, HDAC2, HDAC6, and H3K9Ac allowed for a clear differentiation between normal and neoplastic tissues in multivariate models. ROC curve analysis further confirmed high sensitivity and specificity in distinguishing malignant from normal tissues, highlighting their potential as diagnostic or prognostic biomarkers.

Several factors can influence the prognosis of dogs with mammary tumors, including tumor subtype, tumor size, histological grade, and treatments administered [5, 34]. The observation of aberrant enzyme expression in canine mammary neoplasms encourages further research into other associations related to prognosis and epigenetic alterations.

The therapeutic relevance of these findings is noteworthy. HDACi, particularly those targeting HDAC6, have shown promise in preclinical studies for various cancers, including breast, prostate, and lymphomas [10, 47]. In veterinary medicine, early-phase trials suggest that these compounds may be well tolerated in dogs and merit further investigation [48, 49].

Cell lines from various types of canine cancers have been studied with HDACi, and the results indicated antitumor responses to these drugs [47, 49, 50]. Therefore, examining the dynamics of these aberrant expressions in different neoplastic tissues complements in vitro results and assists in identifying patients who could genuinely benefit from these therapies.

In conclusion, the differential expression of histone acetylation markers and deacetylase enzymes between normal and neoplastic canine mammary tissues suggests that epigenetic dysregulation is involved in tumor development and progression. In particular, the negative association between HDAC6 and H3K9 acetylation supports its potential as a therapeutic target. These findings provide a foundation for future studies investigating HDACi as part of the treatment strategy for canine mammary tumors.

## Figures and Tables

**Table 1:** Antibodies used in the immunohistochemistry technique for the expression of acetylated histones and deacetylase enzymes.

Antibody^∗^	Clone	Origin	Type	Dilution	Antigenic recovery
Anti-acetyl histone H3 (Ac-Lys9)	AH3-120	Rat	Monoclonal	1:100	Citrate buffer (pH 6)
Anti-acetyl histone H4 (Ac-Lys12)	SAB4200353	Rabbit	IgG fraction	1:200	Citrate buffer (pH 6)
Anti-HDAC1	AV38530	Rabbit	IgG fraction	1:75	EDTA ± buffer (pH 9)
Anti-HDAC2	HDAC2-62	Rat	Monoclonal	1:500	Citrate buffer (pH 6)
Anti-HDAC6	AV31451	Rabbit	Isolated	1:500	Citrate buffer (pH 6)

*Note:* ± Ethylenediamine tetra acetic acid; all antibodies were incubated for two hours at 22°C.

^∗^Sigma-Aldrich.

**Table 2:** Results of the exploratory multivariate factor analysis and Wilcoxon–Mann–Whitney test for each antibody (H3K9Ac, H4K12Ac, HDAC1, HDAC2, and HDAC6) within each experimental group (G1: normal tissue, G2: adenoma, G3: simple carcinoma, and G4: metastatic carcinoma).

Antibody		G1		G2		G3		G4	
		F1	F2	F1	F2	F1	F2	F1	F2
H3K9Ac		0.10	**0.68**	0.04	**0.85**	**0.73**	−0.06	0.29	**0.79**
H4K12Ac		**0.86**	0.31	0.01	**0.68**	0.58	0.20	0.58	0.09
HDAC1		**0.91**	−0.12	**0.82**	0.05	0.30	**0.80**	**0.79**	−0.34
HDAC2		−0.10	−0.45	0.38	−0.42	−0.24	**0.77**	**0.75**	0.21
HDAC6		0.09	**0.88**	**−0.79**	0.01	**−0.61**	0.15	0.32	**−0.87**

Variance (%)		32	31	29	27	28	26	35	30

Interpretation		H4K12Ac	H3K9Ac	HDAC1	H4K12Ac	H3K9Ac	HDAC1	HDAC1	H3K9Ac
		HDAC1	HDAC6	HDAC6	H3K9Ac	HDAC6	HDAC2	HDAC2	HDAC6

Wilcoxon–Mann–Whitney test		0.41	< 0.001	0.005	0.067	0.051	< 0.001	< 0.001	0.082
*p* value									

Subgroups	A	−0.01 a	−0.69 a	0.70 a	−0.50 a	−0.09 a	−0.70 a	−0.53 a	0.32 a
	B	−0.01 a	1.20 b	−0.51 b	0.36 a	0.10 a	0.77 b	1.05 b	−0.63 a

*Note:* Bold values indicate factor loadings (≥ 0.50), deemed relevant for interpreting Factors 1 and 2.

**Table 3:** Results from the exploratory factor analysis, ANOVA, and ROC curve for histone H3 (H3K9Ac) and histone deacetylase enzymes (HDAC1, HDAC2, and HDAC6) in normal mammary tissues (G1), simple adenomas (G2), simple carcinomas (G3), and metastatic simple carcinomas (G4) in female dogs.

		Factor 1	Factor 2
H3K9Ac		0.13	**0.76**
HDAC1		**0.80**	0.05
HDAC2		**0.77**	0.05
HDAC6		0.01	**−0.80**

Variance (%)		31.15	30.74

Interpretation		HDAC2 e HDAC1	H3K9Ac e HDAC6

ANOVA		0.002	< 0.001
*p* value			

**Multiple comparisons of means by group**			
	***n***		

Control (G1)	11	0.94 ± 0.55_a	0.97 ± 0.59_a
Adenoma (G2)	19	−0.10 ± 0.82_b	0.35 ± 0.80_ab
Simple carcinoma (G3)	46	−0.02 ± 0.99_b	0.24 ± 0.98_b
Metastatic carcinoma (G4)	15	−0.52 ± 1.09_b	−0.41 ± 1.01_b

**Analysis ROC**		**Cutoff value (Sp/Se)**	

G2 vs G1		0.1 (68.4/100)	0.9 (73.7/72.7)
G3 vs G1		0.4 (73.9/90.9)	0.9 (91.3/72.7)
G4 vs G1		0.4 (90.9/80.0)	0.9 (72.7/93.3)

*Note:* Bold values indicate factor loadings (≥ 0.50), deemed relevant for interpreting Factors 1 and 2.

## Data Availability

The data that support the findings of this study are available from the corresponding author upon reasonable request.
